# Paraneoplastic Hyperfibrinolysis in Oesophageal Adenocarcinoma: A Case Report

**DOI:** 10.7759/cureus.90922

**Published:** 2025-08-25

**Authors:** Yuhui Zhou, William Gelson, Rebecca Brais, Martin Besser

**Affiliations:** 1 Haematology, Addenbrooke's Hospital, Cambridge University Hospitals National Health Service (NHS) Foundation Trust, Cambridge, GBR; 2 Hepatology, Addenbrooke's Hospital, Cambridge University Hospitals National Health Service (NHS) Foundation Trust, Cambridge, GBR; 3 Pathology, Addenbrooke's Hospital, Cambridge University Hospitals National Health Service (NHS) Foundation Trust, Cambridge, GBR

**Keywords:** coagulation, fibrinolysis, hyperfibrinolysis, oesophageal cancer, paraneoplastic process, thromboelastography (teg)

## Abstract

Hyperfibrinolysis is a rare but life-threatening coagulopathy that can occur as a paraneoplastic syndrome. It often remains undetected with routine coagulation tests, requiring specialised diagnostics.

We present a male patient in his 70s with a history of oesophagectomy for oesophagogastric junction adenocarcinoma who presented with dysphagia, epigastric pain, and spontaneous bruising. Laboratory tests revealed elevated D-dimer, low fibrinogen, and a prolonged prothrombin time. Rotational thromboelastometry (ROTEM) revealed a lysis index at 60 minutes of 53%, consistent with hyperfibrinolysis. Extensive investigations, including imaging and bone marrow biopsy, did not show haematologic malignancy or radiological metastatic disease. A transjugular liver biopsy ultimately confirmed occult metastatic oesophageal adenocarcinoma.

This case represents a rare occurrence of primary hyperfibrinolysis linked to oesophageal adenocarcinoma. While the exact pathophysiology remains unclear, it is believed to result from tumour-driven overexpression of fibrinolytic proteins. Given its association with high mortality and poor prognosis, early recognition and intervention are crucial. Standard coagulation tests may fail to detect hyperfibrinolysis, making viscoelastic testing a valuable diagnostic tool. Clinicians should maintain a high index of suspicion for occult malignancy in patients presenting with unexplained bleeding.

## Introduction

Fibrinolysis and coagulation synergise to maintain vasculature patency under normal conditions [1]. Routine coagulation tests are designed to detect hypocoagulability, whereas tests for hyperfibrinolysis or hypercoagulability are the domain of specialist haematology investigations in cases of unexplained bleeding. Acquired bleeding tendencies pose a major diagnostic challenge, and specialist testing may be required where there is a clinical haemorrhagic diathesis unexplained by routine coagulation testing.

Hyperfibrinolysis is an uncommon but potentially fatal paraneoplastic manifestation, most frequently described in association with prostate cancer and less often with other solid tumours such as breast carcinoma and sarcoma [2-4]. To date, no cases linking hyperfibrinolysis to oesophageal adenocarcinoma have been reported in the literature. We present this case to highlight the diagnostic utility of viscoelastic testing, to emphasise the importance of maintaining suspicion for occult malignancy in patients with unexplained bleeding, and to contribute the first documented report of paraneoplastic hyperfibrinolysis in oesophageal adenocarcinoma.

## Case presentation

In October 2023, a Caucasian male in his 70s presented with a two-week history of dysphagia and epigastric discomfort, alongside a one-week history of swelling in his left knee with a reduced range of movement. He reported having received his fourth booster dose of a messenger ribonucleic acid (mRNA) coronavirus disease 2019 (COVID-19) vaccine two weeks prior and described a progressive four-week decline in exercise tolerance. On examination, multiple skin haematomas at varying stages of resolution were noted across the central chest, hips, ankles and feet. Abdominal palpation revealed tenderness in the epigastric region and right upper quadrant.

His medical history was notable for oesophagogastric junction adenocarcinoma, treated with oesophagectomy four months prior to presentation. Histology revealed a poorly to moderately differentiated tumour staged as T3N1R0, indicating invasion through the muscularis propria (T3), metastasis to regional lymph nodes (N1), and no residual tumour at the resection margins (R0). A positron emission tomography (PET-CT) scan approximately one year before presentation demonstrated normal tracer uptake in the liver, spleen, pancreas and kidneys, consistent with the absence of metastatic disease. Neoadjuvant chemotherapy was not indicated, and while adjuvant therapy was discussed, it was not pursued. The chronological sequence of key clinical events is outlined in Table 1, and blood laboratory results on admission are summarised in Table 2.

**Table 1:** Timeline of clinical events Chronological summary of the patient’s presentation, investigations, interventions, and outcomes. All dates are presented relative to admission to preserve anonymity. ROTEM: rotational thromboelastometry

Timepoint	Event
1 year before presentation	Diagnosis of oesophageal adenocarcinoma. Positron emission tomography/computed tomography showed no evidence of metastasis
4 months before presentation	Oesophagectomy (no adjuvant therapy)
4 weeks before admission	Gradual decline in exercise tolerance
2 weeks before admission	Onset of dysphagia, epigastric discomfort, and received 4th COVID-19 booster
1 week before admission	Left knee swelling developed
Admission	Spontaneous bruising; abnormal coagulation profile (low fibrinogen, high D-dimer, ROTEM abnormalities)
Day 4	Retroperitoneal haemorrhage; intensive care admission
Day 7	CT abdomen/pelvis: portal vein thrombosis, no metastases identified
Day 11	Transjugular liver biopsy: occult metastatic oesophageal carcinoma confirmed
Late admission	Progressive bleeding despite therapy; transitioned to palliative care

**Table 2:** Blood laboratory results on admission

Laboratory test	Unit	Value on admission	Reference range	Deviation
Haemoglobin (Hb)	Grams per litre (g/L)	120	Males: 130-180	Low
Platelet count (PLT)	×10⁹ per litre (×10⁹/L)	215	150-400	Normal
White blood cell count (WBC)	×10⁹ per litre (×10⁹/L)	7.3	4.0-11.0	Normal
Lymphocyte count	×10⁹ per litre (×10⁹/L)	0.93	1.0-3.5	Low
Neutrophil count	×10⁹ per litre (×10⁹/L)	7.88	2.0-7.5	High
C-reactive protein (CRP)	Milligrams per litre (mg/L)	39	<5	High
Prothrombin time (PT)	Seconds	16.1	9-13	High
International normalised ratio (INR)	-	1.39	0-1.2	High
Activated partial thromboplastin time (APTT)	Seconds	30.9	22-36	Normal
Fibrinogen	Grams per litre (g/L)	1.3	1.5-4.5	Low
D-dimer	Nanograms per millilitre (ng/mL)	3309	<500	High
Alkaline phosphatase (ALP)	International units per litre (IU/L)	575	30-130	High
Alanine aminotransferase (ALT)	International units per litre (IU/L)	229	10-50	High
Bilirubin	Micromoles per litre (µmol/L)	25	3-17	High
Ferritin	Nanograms per millilitre (ng/mL)	354	Males: 25-350	Normal (upper end)

**Figure 1:**
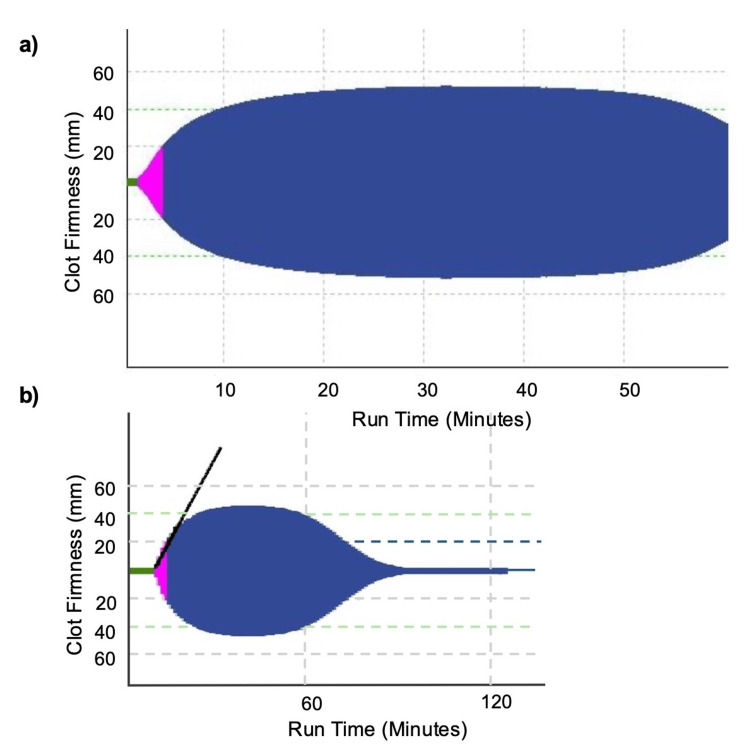
ROTEM EXTEM assay tracing demonstrating hyperfibrinolysis (a) Initial portion of the EXTEM tracing showing clot initiation and early clot firmness development; (b) Extended time scale of the same tracing illustrating marked clot breakdown over time. This figure is provided for illustrative purposes; exact parameter values are reported in Table 3. ROTEM: rotational thromboelastometry; EXTEM: extrinsic thromboelastometry

The full blood count was largely unremarkable, aside from a modest neutrophilia. Coagulation studies showed a normal activated partial thromboplastin time (APTT), but a slightly prolonged prothrombin time (PT) and elevated international normalised ratio (INR). Although these abnormalities were mild in degree, they were clinically significant in the context of active bleeding. D-dimer levels were significantly raised, and fibrinogen levels were notably low. Liver function tests showed a deterioration compared to results from three months prior. Specialist haemostasis investigations revealed a discrepancy between fibrinogen quantity and function. The fibrinogen antigen level was normal at 2.09 grams per litre (g/L) (reference range: 2-4 g/L); however, Clauss fibrinogen activity was abnormally low at 1.03 g/L (reference range: 1.5-4.0 g/L). To further assess clot stability, rotational thromboelastometry (ROTEM) was performed. The lysis index at 60 minutes was markedly reduced at 53% (reference range: 90-100%), consistent with hyperfibrinolysis (Table 3, Figure 1).

**Table 3:** Thromboelastography parameter values Viscoelastic testing was performed using rotational thromboelastometry (ROTEM) with the EXTEM assay. The most clinically relevant abnormality was a markedly reduced LI60, consistent with pathological hyperfibrinolysis. EXTEM: extrinsic thromboelastometry

Parameter	Unit	Value	Reference range
Clotting time (CT)	Seconds (s)	71	38-79
Clot formation time (CFT)	Seconds (s)	155	34-159
Alpha angle (α)	Degrees (°)	60	67-83
Amplitude at 5 minutes (A5)	Millimetres (mm)	32	-
Amplitude at 10 minutes (A10)	Millimetres (mm)	42	-
Amplitude at 20 minutes (A20)	Millimetres (mm)	50	-
Amplitude at 30 minutes (A30)	Millimetres (mm)	52	-
Maximum clot firmness (MCF)	Millimetres (mm)	52	54-72
Area under the curve (AUC)	-	5156	5314-7000
Time to max velocity of clot formation (MAXV-t)	Seconds (s)	146	46-140
Maximum lysis (ML)	Per cent (%)	11	0-15
Lysis index at 30 minutes (LI30)	Per cent (%)	100	94-100
Lysis index at 45 minutes (LI45)	Per cent (%)	97	90-100
Lysis index at 60 minutes (LI60)	Per cent (%)	53	85-100
Maximum velocity of clot formation (MAXV)	Millimetres per minute (mm/min)	8	-
Maximum clot elasticity (MCE)	Dynes per square centimetre (dyn/cm²)	107	86-230
Time to maximum clot firmness (MCF-t)	Seconds (s)	1784	-

**Figure 2:**
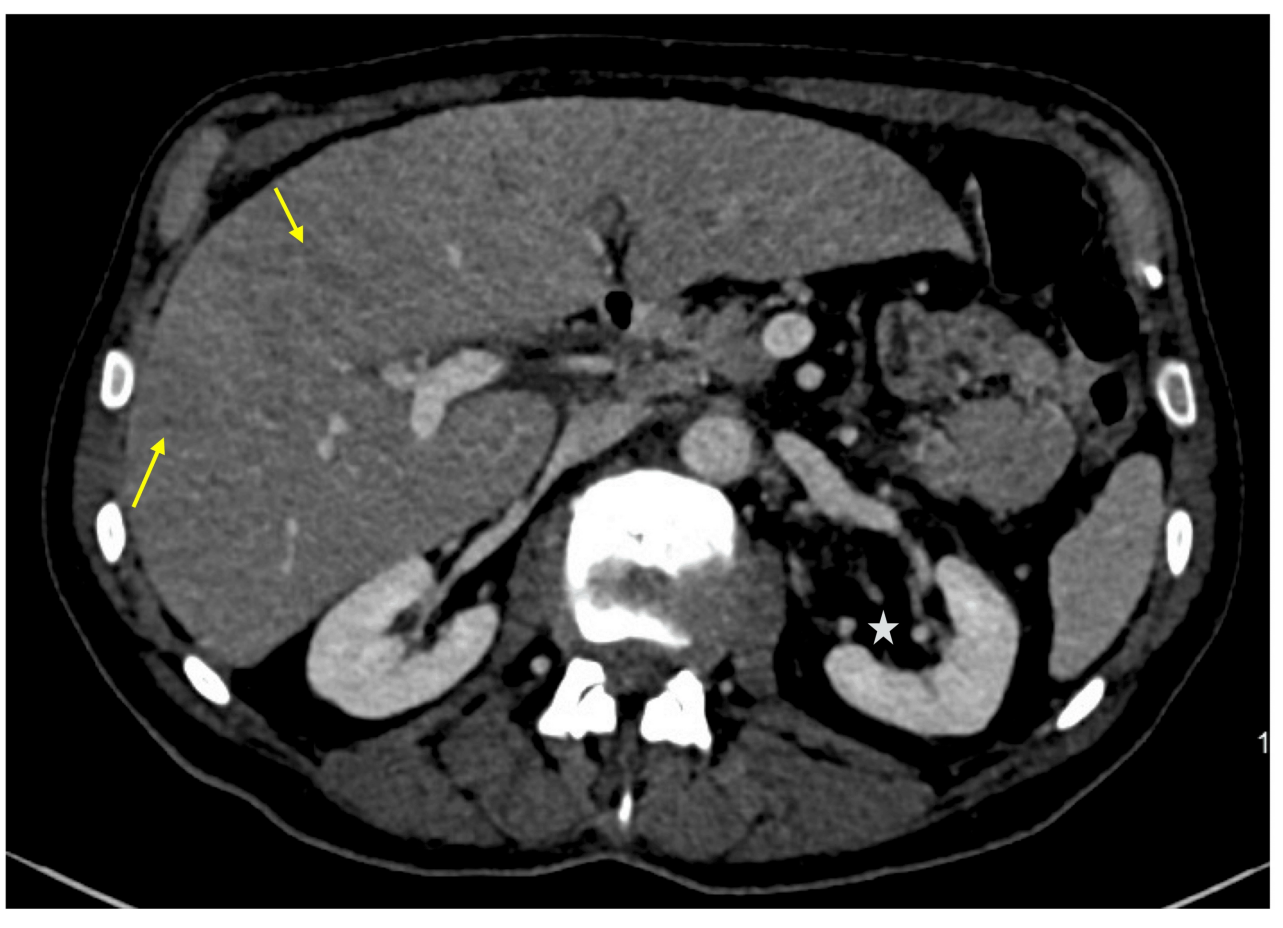
Axial computed tomography (CT) image of the thorax, abdomen, and pelvis showing slight heterogeneity of the liver The arrows point to heterogeneous liver enhancement. The star indicates left-sided retroperitoneal fat stranding.

Potential intra-abdominal causes for these findings were explored using imaging. A computed tomography (CT) scan of the thorax, abdomen, and pelvis revealed features of left-sided retroperitoneal fat stranding and slightly heterogeneous liver enhancement (Figure 2). Additionally, ultrasound Doppler imaging of the liver identified indeterminate hypoechoic regions. Given the significantly elevated D-dimer levels, an ultrasound Doppler scan of the lower limbs ruled out deep vein thrombosis. The swelling in the patient’s left leg was attributed to a haematoma.

Differential diagnosis

The initial differential diagnosis included haematological malignancies, potential cancer-associated disseminated intravascular coagulation (DIC), and unexplained liver dysfunction. Other considerations involved vaccine-induced thrombocytopenia and thrombotic syndrome (VITT), as well as a possible paraneoplastic immune-mediated disruption of coagulation. Haematological malignancy with associated clotting derangement was initially the leading differential diagnosis, given the lack of radiological evidence of solid organ disease. This was closely followed by suspected early-stage massive activation of the clotting cascade in cancer-associated DIC. A bone marrow biopsy was performed, facilitated by cryoprecipitate infusion due to low fibrinogen level. The biopsy revealed no evidence of primary haematological malignancy or bone marrow infiltration by solid organ malignancies. 

Based on initial imaging and prior PET-CT findings, there was no definitive evidence of primary or infiltrative liver disease. However, differentials for deranged liver enzymes included infectious, autoimmune, and vascular causes of liver dysfunction. The only mildly elevated PT, a marker of liver synthetic function, argued against primary liver dysfunction as the cause. The history of a recent COVID-19 vaccination raised the possibility of VITT. However, the patient did not fit the diagnostic criteria established by the Brighton Collaboration [5]. Specifically, the D-dimer level was <4000, the platelet count was >150 × 10⁹/litre, and anti-platelet factor 4 (anti-PF4) antibodies were negative. The following immunological and tumour marker tests were performed. All results were negative or within normal ranges, except for a mildly elevated carcinoembryonic antigen (CEA) (Table 4).

**Table 4:** Immunological and tumour marker tests

Test	Result
Antinuclear antibodies (ANA), including double-stranded DNA (dsDNA), extractable nuclear antigen (ENA), and centromere antibodies	Negative
Glomerular basement membrane antibody (GBM antibody)	Negative
Rheumatoid factor (RF)	Negative
Antineutrophil cytoplasmic antibodies (ANCA)	Negative
Gastric parietal cell antibody	Negative
Smooth muscle antibody (SMA)	Negative
Liver-kidney microsomal antibody (LKM antibody)	Negative
Complement components 3 and 4 (C3/C4)	Normal
Cryoglobulin	Negative
Serum protein electrophoresis (SPEP)	Normal
Carbohydrate antigen 19-9 (CA 19-9)	Normal
Carcinoembryonic antigen (CEA) (Reference range: 0.0 – 5.0 µg/L)	Mildly elevated (6.4)
Total prostate-specific antigen (Total PSA)	Normal

Treatment/ongoing investigations 

**Figure 3:**
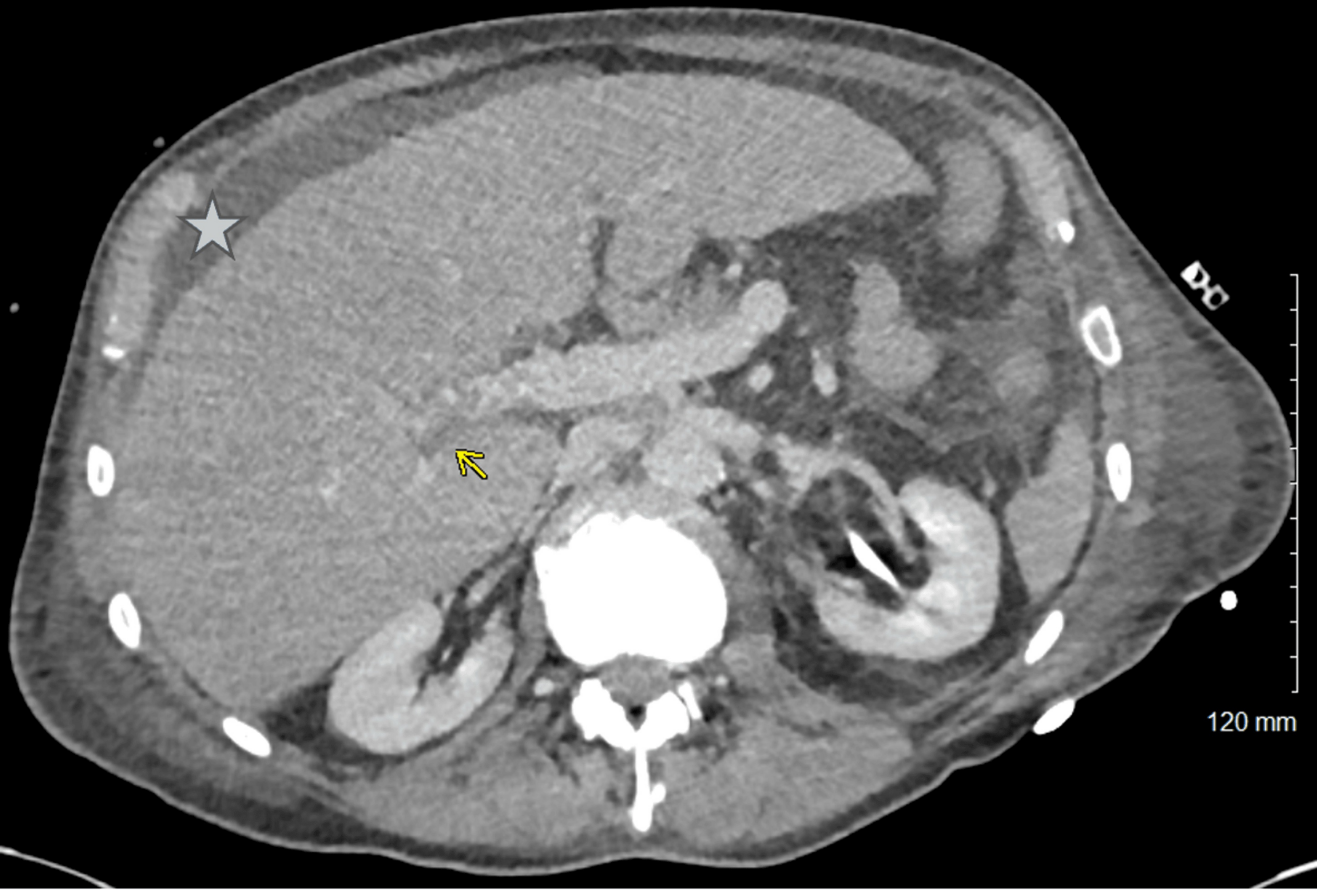
CT abdomen pelvis showing portal vein thrombosis The arrow points to portal vein thrombosis. The star indicates ascites.

On the fourth day of admission, the patient developed a haemodynamically relevant spontaneous retroperitoneal bleed, with a haemoglobin drop from 101 g/L to 57 g/L (reference range: 130 - 180 g/L) within 12 hours, necessitating admission to the intensive care unit (ICU) for blood pressure support. Treatment included cryoprecipitate in combination with tranexamic acid, administered at 1 g three times daily. Tranexamic acid was administered in accordance with institutional haemostasis protocol. Aprotinin was considered, but given its very narrow license for cardiopulmonary bypass surgery, it was not deemed appropriate as first-line therapy. Epsilon aminocaproic acid is not available in the United Kingdom. Concern existed regarding the potential risk of precipitating thrombosis by inhibiting the fibrinolytic process, but this risk had to be balanced against the occurrence of spontaneous massive haemorrhage despite fibrinogen replacement therapy. Given the patient’s acute haemodynamic instability and active bleeding, haemostatic stabilisation was prioritised. Low-dose heparin was initiated to offset the prothrombotic risk associated with tranexamic acid, compounded by the patient’s malignancy history and immobility. A higher dose was considered desirable but regarded as unsafe in the setting of life-threatening haemorrhage; instead, a plan was made to uptitrate the dose gradually as tolerated, with the goal of reaching prophylactic anticoagulation. Following a deterioration in liver function tests, a CT scan was performed, revealing a portal vein thrombosis but no evidence of recurrent or metastatic cancer (Figure 3). 

**Figure 4:**
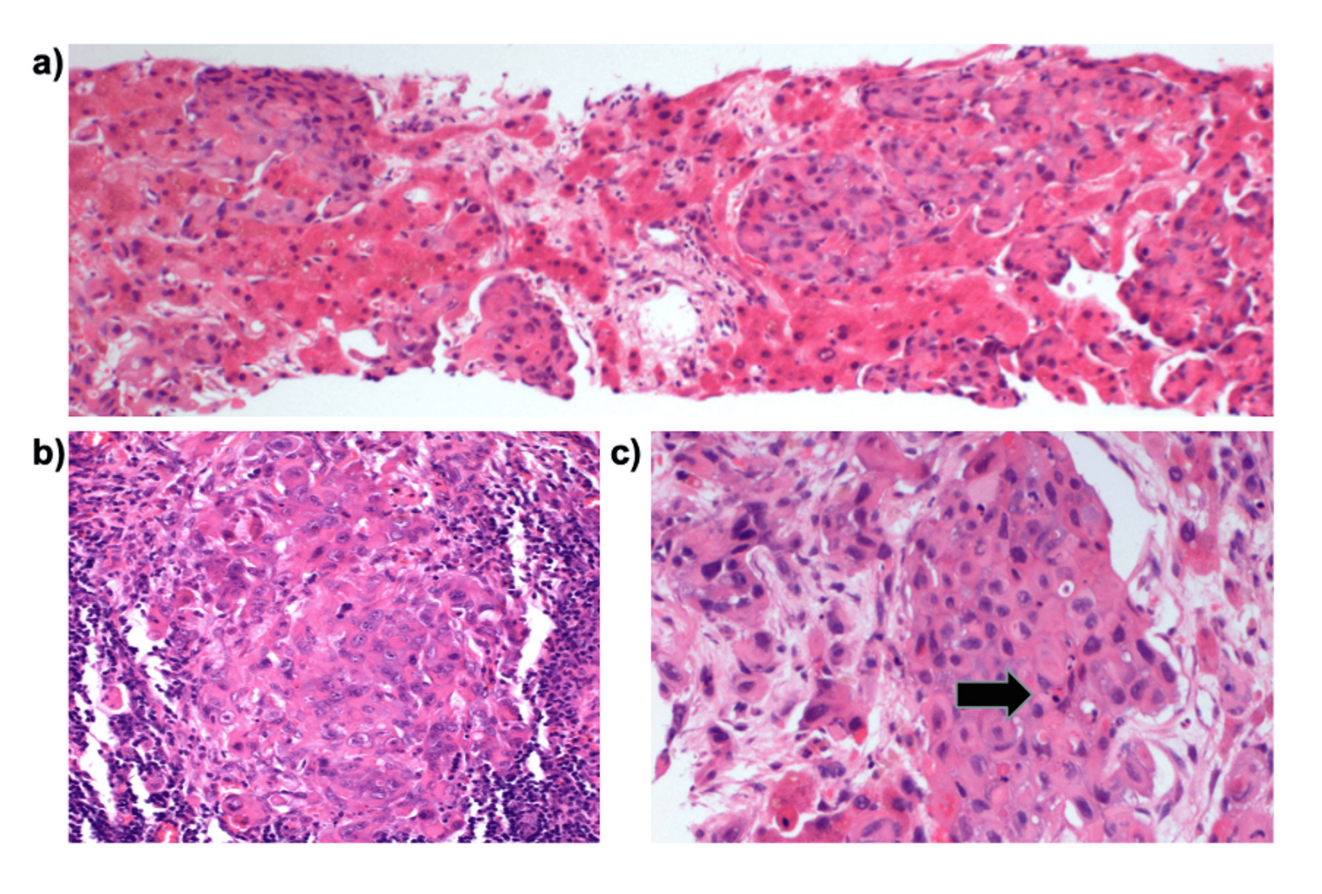
Histology of liver biopsy and prior lymph node biopsy (a) Liver biopsy showing sinusoidal infiltration by poorly differentiated carcinoma (H&E stain); (b) Lymph node metastasis from prior oesophageal adenocarcinoma with similar morphology (H&E stain); (c) Signet ring cells identified in the liver specimen (H&E stain). H&E: haematoxylin and eosin

On the 11th day of admission, a transjugular liver biopsy was conducted. Microscopically, the specimen showed extensive sinusoidal infiltration by a poorly differentiated carcinoma (Figure 4a), with the presence of signet ring cells (Figure 4c). Morphological features bore resemblance to areas of the tumour observed in the resected specimen of lymph node metastases from the patient’s previous oesophageal carcinoma (Figure 4b).

Outcome

Unfortunately, the patient’s condition continued to deteriorate, with uncontrollable bleeding and haemodynamic instability. Following discussions between the patient’s family and the clinical team, a joint decision was made to transition to symptom-focused palliative care. The patient passed away shortly thereafter.

## Discussion

The study of fibrinolysis dates to the anatomist and physician Morgagni in the 18th century, who observed that blood did not always coagulate after sudden death [6]. By the 19th century, researchers recognised that clot formation and breakdown involved enzymatic digestion, with the term "fibrinolysis" first appearing in 1893 [7]. Macfarlane described post-surgical hyperfibrinolysis and detection methods [8]. The terms “plasminogen” and “plasmin,” introduced by Christensen and MacLeod in 1945, were soon widely adopted [9]. Hyperfibrinolysis was later observed in conditions such as pregnancy-associated toxaemia, burns, haemorrhage, and barbiturate poisoning. Ratnoff documented the first case linked to pancreatic carcinoma during surgery in 1952 [10]. Euglobulin lysis time (ELT), as described in early fibrinolysis studies, quantifies fibrinolytic activity by measuring the time taken for a precipitated euglobulin fraction of plasma-containing plasminogen and activators but excluding inhibitors such as α2-antiplasmin-to undergo enzymatic clot lysis, thereby assessing intrinsic fibrinolytic potential [11]. It is not in routine use due to its long turnaround time, exclusion of key fibrinolysis inhibitors, and inability to provide real-time clot dynamics.

Viscoelastic testing (thromboelastography {TEG} or ROTEM), developed by Hartert in 1948, is a viscoelastic haemostatic assay that simulates sluggish venous flow [12]. It utilises an oscillating cup containing blood, immersed in a sensor shaft, to evaluate all stages of clot initiation, formation, stability, strength and dissolution in whole blood [13]. Indices such as the lysis index assess clot stability and the rate of fibrinolysis at specified intervals after clot formation. The sensitivity of viscoelastic testing is unclear, but where it is detected, it produces a pathognomonic trace and is associated with high mortality [14,15].

Pathological hyperfibrinolysis can be classified as primary or secondary. Differentiating between primary and secondary hyperfibrinolysis can be difficult. Primary hyperfibrinolysis results from an imbalance between pro-fibrinolytic factors, such as plasminogen activators, and anti-fibrinolytic factors, including PAI-1 and TAFI. Malignancies may disrupt the fibrinolytic process, contributing to hyperfibrinolysis [16]. Secondary hyperfibrinolysis, on the other hand, arises from massive activation and consumption of haemostatic factors, followed by excessive fibrinolysis, as seen in DIC [3]. DIC is known to be associated with metastatic cancer and is more common than hyperfibrinolysis. It occurs in 10-15% of solid malignancies and 15% of acute leukaemia [17]. By contrast, the true incidence of primary hyperfibrinolysis in solid tumours is unknown, but available reports suggest it is exceedingly rare.

In this case, the initial findings of elevated D-dimer and low fibrinogen activity could suggest either hyperfibrinolysis or DIC. However, the isolated low lysis index at 60 minutes on TEG strongly indicated primary hyperfibrinolysis. The borderline-normal PT, APTT, and platelet count, combined with the absence of hypercoagulability or hypocoagulability on TEG, effectively ruled out early or late-stage DIC. The decision to defer repeat imaging or biopsy was driven by the need to correct life-threatening coagulopathy before further diagnostics. Although earlier imaging or biopsy might have identified occult metastatic disease sooner, this would likely not have altered immediate management or overall outcome, given the rapid clinical deterioration.

The liver synthesises most pro- and anti-fibrinolytic factors, and liver disease can lead to various fibrinolytic states. Current understanding suggests that liver disease results in a state of rebalanced haemostasis due to alterations in both pro- and anti-haemostatic factors, including reduced levels of alpha-2 antiplasmin and plasminogen, as well as increased levels of tissue plasminogen activator (tPA) and PAI-1. The balance may tip with the advancement of liver disease, explaining the heightened risk of both bleeding and thrombotic complications [18]. Acute liver disease, by contrast, is generally associated with hypofibrinolysis [18]. In this case, the patient presented with acute liver enzyme derangement and a bleeding phenotype secondary to hyperfibrinolysis, without any prior history of liver disease.

The pathophysiology of cancer-associated hyperfibrinolysis is poorly understood. It has been proposed that cancer cells may produce proteins, such as urokinase plasminogen activator (u-PA), which disrupt the balance between pro- and anti-fibrinolytic drivers [19]. For instance, u-PA and its receptor are expressed at significantly higher levels in prostate cancer tissue (the most common tumour type associated with hyperfibrinolysis) compared to normal prostate tissue and are implicated in tumour invasion and metastasis [20]. Similarly, the urokinase plasminogen activator system (uPAS) is strongly associated with the metastasis of gastric and oesophageal cancers, likely through the activation of plasmin, which facilitates the breakdown of extracellular matrix proteins to aid tumour invasion. Tumour-associated stromal cells, including macrophages, also express key components of the uPAS [21]. Overexpression of uPAS is correlated with poorer disease-free and overall survival in gastric and oesophageal cancer, with hazard ratios of 2.21 (95% confidence interval (CI) 1.71-2.86) for overall survival and 1.78 (95% CI 1.40-2.26) for disease-free survival [22].

Hyperfibrinolysis has been described in association with other solid tumours, including prostate, breast, and rhabdomyosarcoma [2-4,23]. Reported cases are scarce and vary widely in detail. A 2021 systematic review of 12 reports (21 patients) of solid cancer-associated hyperfibrinolysis found prostate cancer to be the most frequent (≈76%) [2]. However, differences in tumour type, stage, laboratory work-up, and treatment limit direct comparison. Table 5 summarises these published reports, highlighting both the rarity of cancer-associated hyperfibrinolysis and the heterogeneity of management strategies and prognosis. In oesophageal adenocarcinoma, five-year survival for T3 disease is estimated at only 20% [24]. Against this background, the recurrence of cancer and the development of hyperfibrinolysis in our patient highlight the clinical significance of this uncommon haemostatic complication.

**Table 5:** Summary of published reports of hyperfibrinolysis in solid tumours Reported cases include single case reports of prostate cancer [23], breast cancer [3], and rhabdomyosarcoma [4], as well as a systematic review of 12 reports encompassing 21 patients, in which prostate cancer accounted for approximately 76% of cases [2].

Reference	Year of publication	Type of study	Number of patients	Type(s) of solid organ cancer	Management	Outcome
Winther-Larsen et al. [2]	2021	Systematic review of case reports	21 (12 reports)	Mainly prostate cancer (16); also breast (2), lung (1), melanoma (1), cancer of unknown primary (1)	Blood products (fibrinogen replacement, plasma, platelets, red blood cells), antifibrinolytics, haemodynamic/ICU support, oncologic therapy (varied by tumour type)	~67% died within 6 months
Sharma [23]	2021	Case report	1	Prostate cancer	Blood products (plasma), oncologic therapy (hormonal therapy, radiotherapy)	Bleeding resolved; patient discharged stable and alive at 4-month follow-up
Lu et al. [3]	2023	Case report	1	Breast cancer	Blood products (fibrinogen replacement), tranexamic acid, oncologic therapy (chemotherapy)	Bleeding controlled; underlying metastatic breast cancer with poor overall survival expectation
Taylor et al. [4]	2024	Case report	1	Rhabdomyosarcoma	Blood products (cryoprecipitate, platelets, red blood cells), aminocaproic acid, oncologic therapy (chemotherapy)	Haematuria resolved; coagulation normalised; patient continued oncologic treatment with good short-term response

There is no systematic data comparing outcomes in patients with solid tumours who present with or without detectable signs of hyperfibrinolysis. In our case, the patient’s oesophageal cancer was surgically resected, and neither initial nor subsequent imaging showed definitive evidence of metastatic disease until a liver biopsy was performed. To our knowledge, this is the first reported case of primary hyperfibrinolysis linked to oesophageal adenocarcinoma.

## Conclusions

In summary, a high degree of clinical suspicion for disseminated cancer or leukaemia is crucial when diagnosing hyperfibrinolysis, particularly in the context of malignancy. As demonstrated in this rare case of oesophageal adenocarcinoma, hyperfibrinolysis can present as an early paraneoplastic phenomenon, even in the absence of evident metastasis. Diagnosis is often challenging due to the low sensitivity of routine coagulation tests and the overlap of biomarkers with other conditions such as DIC. In patients with a history of cancer presenting with hyperfibrinolysis, thorough investigations are essential. Viscoelastic testing can provide valuable insights; however, the negative predictive value of a normal trace remains uncertain.
